# The humanitarian–development–peace (triple) nexus: a typology and critical reflections

**DOI:** 10.1111/disa.70067

**Published:** 2026-07-23

**Authors:** Rodrigo Mena

**Affiliations:** ^1^ International Institute of Social Studies Erasmus University Rotterdam The Netherlands

**Keywords:** adaptive implementation, aid coordination, humanitarian–development–peace, institutional strategy, integrated programming, nexus typology, organisational strategies, triple nexus

## Abstract

This article explores how the humanitarian–development–peace (HDP) nexus (also known as the triple nexus) is operationalised, moving beyond policy discourse to implementation on the ground. Drawing on a systematic literature review, programme analysis, and field‐based observation, it proposes three operational approaches to the HDP nexus, integrated, nexus‐sensitive, and coordinated, which often appear in hybrid forms. Building on Paul Howe's (2019) ‘bundles and arrays’ framework, the typology moves the focus from individual project design to broader organisational strategies, institutional mandates, contextual realities, and trade‐offs shaping nexus practice. The analysis situates this within a decade of nexus discourse and implementation, addressing current pressures such as shrinking aid budgets and the integration of climate and displacement agendas, while examining their implications for operational practice. Rather than pursuing a universal model, the paper argues that effective practice resists standardisation, emerging instead from negotiation across shifting mandates, evolving priorities, and diverse contexts, offering a typology to guide context‐specific operational choices.

## 1 INTRODUCTION

The humanitarian–development–peace (HDP) nexus (also known as the triple nexus) promises to bridge long‐standing divides among emergency relief, long‐term development, and peacebuilding, mostly in fragile and conflict‐affected settings. In policy discourse, it is presented as a way of translating this ambition into practice, such as through programmes that combine emergency food aid, livelihood recovery, and local dialogue to meet urgent needs, restore economic activity, and reduce tensions over resources.

Yet, a decade after its endorsement by the United Nations (UN) and the Organisation for Economic Co‐operation and Development (OECD), and despite its place in donor strategies, policy frameworks, and institutional plans (see, for example, Dalrymple and Swithern, 2019; OECD, 2019; Swithern and Schreiber, 2023; Bunse and Delgado, 2024; Ansell, 2025; Tinapp and Mehou, 2025), the triple nexus remains more slogan than practice: approaches vary widely, transformative impact is uneven, and implementation is politically contested and only partially understood. What does the nexus look like on the ground, and why do some approaches gain traction while others stall?

This article addresses these questions by examining how the HDP nexus is operationalised and by proposing a typology that captures how it is interpreted and enacted. It identifies three main approaches, integrated, nexus‐sensitive, and coordinated, which often appear in hybrid forms. Together, they show the nexus as plural and flexible, while illustrating how organisations navigate competing mandates, funding constraints, and political risks. The typology reveals the nexus' strengths, limitations, and conditions for success, reflecting distinct institutional logics, trade‐offs, and operational constraints. It is intended as a reflective tool to help organisations and practitioners assess which form of nexus engagement is realistic and appropriate in specific locations, rather than as a prescriptive template.

This matters because, in a context of increasing pressure to deliver more coherent and efficient responses with fewer resources, understanding how different nexus models function is critical for both analytical clarity and operational decision making. To examine this systematically, the methodology underpinning this study, presented in detail in section three, draws on a meta‐analysis of nearly a decade of research into and engagement with the HDP nexus (2016–25), combining a systematic literature review with insights from programme analysis and field‐based observation.

The article makes three main contributions to current debates on the HDP nexus. First, it moves beyond generic references to ‘doing the nexus’ by conceptualising three empirically‐grounded operational approaches, each with characteristic assumptions, risks, and opportunities. Second, it extends Howe's (2019) ‘bundles and arrays’ framework: whereas Howe offers a programme‐level lens for structuring nexus interventions, the typology proposed here shifts to organisational and systemic dimensions, investigating how actors structure strategies, negotiate mandates, and adapt to political, operational, and resource constraints. Third, it draws out practical implications for donors, international organisations, and local actors by identifying the context conditions under which integrated, nexus‐sensitive, or coordinated approaches are more feasible, and by exploring how to navigate transitions and hybrid forms in practice.

The analysis is particularly timely given changing priorities regarding defence spending, shrinking humanitarian budgets, and the persistent nature of protracted and politically complex crises. Expectations of the triple nexus are intensifying (seamless coordination, pooled multi‐year funding, and simultaneous delivery of humanitarian, development, and peace outcomes) while the gap between ambition and reality remains stark. At the same time, the nexus has become a vehicle for integrating other agendas, including climate change, displacement, and localisation, often without adequate attention to power dynamics or the operational trade‐offs they entail. Understanding of the practical configurations, limitations, and tensions of nexus implementation is therefore urgent.

Ultimately, the article advances current debates on the HDP nexus by moving beyond the systematisation of existing knowledge to provide a forward‐looking analytical tool for reflexive practice. Rather than prescribing how the nexus should be implemented, it asks what kinds of nexus are possible, under which conditions, and with what implications.

The next section reviews the evolution of nexus thinking and key critiques. Section three outlines the methodology. Sections four and five develop the typology and examine its operational implications. The final sections, six and seven, reflect on its relevance for current debates and future practice.

## 2 FRAMING THE NEXUS: CONCEPTS, CRITIQUES, AND CONTRADICTIONS

Efforts to connect aid sectors, such as humanitarian and development actions, are not new. For decades, practitioners and policymakers have questioned the artificial (although oftentimes useful and needed) separation between emergency relief and long‐term development assistance.

As protracted crises became more common and relief and development interventions increasingly overlapped, frameworks such as linking relief, rehabilitation, and development (LRRD) and the ‘relief–development continuum’ sought to blur these boundaries in the late 1990s and early 2000s, promoting sequencing and integration across sectors (Buchanan‐Smith and Fabbri, 2005; Otto and Weingärtner, 2013; Mosel and Levine, 2014). While these approaches made important conceptual and operational contributions, they were criticised for being overly linear and insufficiently attentive to local realities, as well as for sidelining issues related to peace and politics (Hilhorst, 2007; van Dijkhorst, 2013; Mosel and Levine, 2014).

The HDP nexus builds on this legacy but extends it by explicitly incorporating peace alongside humanitarian and development work. The basic proposition is that gains achieved by humanitarian and development interventions are unlikely to be sustainable, and can be rapidly reversed, in the absence of some form of peace. The triple nexus gained formal traction following the World Humanitarian Summit of 2016, where peace was promoted as necessary for reducing humanitarian need and achieving the Sustainable Development Goals (SDGs) (UN OCHA, 2017; Howe, 2019). It was further consolidated following the release of the *DAC Recommendation on the Humanitarian–Development–Peace Nexus* by the OECD's Development Assistance Committee in 2019, reflecting a growing consensus that crises are multidimensional, protracted, and deeply political (OECD, 2019; see also Brown and Mena, 2021). In theory, the nexus aims to improve coordination, reduce duplication, and foster more sustainable outcomes. In practice, its application has revealed diverse challenges and contradictions.

Importantly, framing the nexus as a ‘new’ innovation risks overlooking the long‐standing practices of local organisations, frontline responders, and communities. For many of these actors, working across humanitarian, development, and peace dimensions has been a necessity rather than a designed strategy; an everyday reality rather than a branded framework (Mena and Hilhorst, 2022; Brown, Mena, and Brown, 2024). From their perspective, the HDP nexus is less a novel agenda than a belated recognition of intersectional practices they have long carried out and advocated for acknowledgement. This shift in vantage point, from external narratives of innovation to lived experiences of response, highlights that in many contexts, the nexus does not need to be ‘introduced’; it already exists in local ways of working, even if it has not been labelled as such.

This resonates with the findings of Brown (2025) and Vuijk (2025) who show how national non‐governmental organisations (NGOs) in South Sudan have long operated across humanitarian, development, and peace domains out of pragmatic necessity rather than because of donor‐driven initiatives. Similarly, other studies underline the crucial importance of engaging local organisations and communities in shaping and implementing nexus approaches (Barakat and Milton, 2020; Volkdal, 2024; Plesner Volkdal, 2024). These perspectives complicate the notion of the HDP nexus as a top‐down policy tool and point to the need for a more grounded understanding of how ‘nexus‐like’ practices already function.

At the level of international policy, the HDP nexus is often described as a coordination and planning model aimed at achieving collective outcomes through joint analysis, cross‐sectoral coherence, and mutual reinforcement of humanitarian, development, and peace efforts, frequently at the level of a specific geographic area (Südhoff, Hövelmann, and Steinke, 2020). This vision is reflected in donor strategies and inter‐agency guidance (Poole and Culbert, 2019; OECD, 2019; Ansell, 2025). Yet, there is no single agreed interpretation of what the nexus entails. Instead, its application reflects a range of institutional perspectives, capacities, and priorities, giving rise to several recurring critiques and concerns.

While not exhaustive, five sets of concerns are particularly salient and are relevant for the typology developed in section four:

1. **Conceptual ambiguity, especially around ‘peace’:** persistent uncertainties in the definition and operationalisation of the triple nexus (particularly concerning the ‘peace’ component) risk reducing complex political challenges to technical fixes (Brown, Mena, and Brown, 2024). ‘Peace’ may refer to community cohesion, conflict sensitivity, or everyday safety (‘small p’), or to formal political processes, state building, and structural transformation (‘big P’); although distinct concepts, these broadly resonate with Galtung's (1969) distinction between negative peace (the absence of direct violence) and positive peace (the presence of conditions that address structural and cultural violence) (see also IASC, 2020; Redvers and Parker, 2020). The choice of meaning can have significant operational consequences, shaping which nexus approach actors consider as viable. In some cases, peace programming overlaps with security and stabilisation agendas, raising political sensitivities and challenging the humanitarian principles of neutrality, impartiality, and independence (Lie, 2020; Sommardahl, 2025; Südhoff, 2025). Where the state is a party to the conflict, these tensions intensify and can complicate joint action or undermine humanitarian legitimacy.
2. **Donor‐driven framing and limited local ownership:** structural critiques focus on how the agenda has been largely shaped by the priorities of the so‐called Global North and by multilateral planning processes that may fail to reflect the realities, agency, and institutional diversity of local actors (Dalrymple and Swithern, 2019; Nguya and Siddiqui, 2020; Cochrane and Wilson, 2023). This reinforces the point made above: many local organisations were already working in ‘nexus‐like’ ways before the triple nexus was codified. When nexus frameworks are imposed from outside, they risk instrumentalising local actors or subordinating their priorities to geopolitical or donor logics.
3. **Fragmented funding and conflicting mandates:** competition between sectors, inflexible funding, and competing mandates can marginalise local leadership and limit pooled or adaptable resources (Lie, 2020; Nguya and Siddiqui, 2020; Redvers and Parker, 2020). Even when donors endorse the HDP nexus rhetorically, funding streams often remain siloed, with separate humanitarian, development, and peace envelopes subject to different rules, time frames, and risk appetites (Dalrymple and Swithern, 2019; Rey, Abellán, and Gómez, 2022). These constraints can marginalise local leadership and make it difficult to sustain integrated or longer‐term approaches, particularly in volatile environments.
4. **Internal organisational barriers:** within international organisations, siloed structures, weak cross‐sectoral leadership, high staff turnover, and performance systems prioritising donor compliance over collective impact all hinder nexus implementation. Such operational complexity is exacerbated by multidimensional impact pathways that resist linear measurement and challenge standard monitoring, evaluation, and learning (MEL) systems (Howe, 2019; Südhoff, Hövelmann, and Steinke, 2020; Steinke, 2025). Even when personnel are committed to nexus thinking, institutional incentives and bureaucratic routines can pull them back into sector‐specific logics.
5. **Discourse–practice gap:** high‐level frameworks present the HDP nexus as a coordination tool focused on shared outcomes, but operationalisation is uneven, contested, and shaped by institutional constraints, actor interpretations, and highly context‐specific practices (see, for example, Howe, 2019; Lie, 2020; National HDP Nexus Task Force, Cameroon, 2023; Talisuna et al., 2023; Sommardahl, 2025). Relatedly, there are calls to temper expectations of what the nexus can achieve, noting that climatic, security, and institutional dynamics can limit or distort its impact (Cochrane and Wilson, 2023; Plesner Volkdal, 2024).

Across all of the above is a common thread: politics. The politics of the HDP nexus cannot be separated from the approach itself. Driven primarily by donors and multilateral actors, often as a condition for funding alignment or programming eligibility, the nexus can serve geopolitical or state‐building agendas alongside humanitarian, development, and peace objectives (Dalrymple and Swithern, 2019; Cochrane and Wilson, 2023). While promoted as a coordination instrument, the nexus is never outside the power dynamics and political economy that shape the aid system. Thus, while the ‘P’ in the triple nexus usually stands for peace (in its many variants, such as peacebuilding, conflict sensitivity, or social cohesion), it is equally important to consider what might be called the transversal ‘P’: *politics*. In every component—humanitarian, development, and peace—and in the links between them, politics are embedded, influencing, stretching, and constraining interventions.

Lastly, nexus practices are also affected by intersectional factors like gender, race, class, and locality, which impact who participates in decision making, whose knowledge is valued, and who bears the risks of experimentation. While this article focuses primarily on institutional and operational dynamics, these intersectional dimensions remain crucial and are revisited later. Building on the abbreviated conceptual history and critiques outlined above, section four proposes a typology of three main HDP nexus approaches. This is intended as a lens with which to analyse, compare, and adapt nexus practice to specific contexts, while keeping these underlying tensions and power relations in view. But first, a look at the methodology.

## 3 METHODOLOGY AND SOURCES

The analysis in this article is based on meta‐level engagement with the HDP nexus over nearly a decade (2016–25), combining a systematic review of written sources with insights from programme analysis and field‐based observation. The core empirical foundation is a structured review of more than 120 documents published between 1993 and 2025, including peer‐reviewed journal articles, policy reports, organisational guidelines, and grey literature addressing nexus implementation. The starting point in the early 1990s reflects the emergence of earlier linkage frameworks, such as LRRD and the relief–development continuum, which prefigure key elements of the contemporary nexus debate and provide an essential historical baseline.

The reviewed materials were identified through iterative database searches using terms such as ‘triple nexus’, ‘HDP nexus’, ‘relief–development linkages’, ‘LRRD’, and ‘peace–development nexus’, complemented by citation tracing of key publications and assessment of policy and guidance documents produced by major donors, UN agencies, and international NGOs. The documents were analysed through thematic reading, with particular attention paid to how different actors describe nexus objectives, operational modalities, institutional constraints, and perceived strengths and weaknesses. These dimensions served as initial analytical themes, allowing the identification of recurring patterns of discourse and practice across contexts.

The literature review is supplemented by insights from previous research projects and professional engagements in crisis‐affected settings, including Afghanistan, Colombia, South Sudan, Ukraine, and Yemen. These projects involved qualitative interviews and discussions with the staff of international and national NGOs, UN agency personnel, and donor representatives, as well as direct observation of programming and coordination processes. While those data were collected for other studies and some have been reported elsewhere (Brown and Mena, 2021; Brown, Mena, and Brown, 2024), they inform the interpretation and illustration of nexus practices in this article. The typology is thus not derived from a single case study, but from a cross‐case synthesis of how organisations describe and experience the HDP nexus in different operational environments.

While nexus practices are highly diverse and context‐specific, the comparative thematic analysis revealed that most documented approaches tend to cluster around four recurring modes of operationalisation. These clusters were identified inductively through cross‐case comparisons of organisational strategies, mandate negotiations, funding arrangements, and responses to political and operational constraints (as analytical categories). A second round of analysis showed that what were originally identified as distinct systems‐ *and* governance‐oriented approaches were in fact different ways of seeing the same integrated nexus strategy, and hence they were combined. The resulting typology of three nexus approaches (integrated, nexus‐sensitive, and coordinated) should thus be understood as an analytical synthesis of recurrent patterns in organisational practice and discourse, rather than as a predefined classification or a statistically representative mapping of all nexus initiatives.

The aim of this methodological approach is not to evaluate the effectiveness of individual programmes, but to generate an analytically robust framework that captures the dominant ways in which the HDP nexus is interpreted and enacted in practice, along with the trade‐offs they entail. The cases referenced throughout the article, such as nexus initiatives in Cameroon, Ethiopia, South Sudan, or Ukraine, are used illustratively to ground the typology in concrete examples. The claims advanced here should therefore be treated as analytically‐grounded lessons learned based on converging patterns across sources, rather than as universal or prescriptive conclusions.

## 4 A TYPOLOGY OF THE NEXUS: MANY SHADES, ONE COLOUR

Despite the prominence of the HDP nexus in policy discourse, its implementation remains uneven and highly context‐dependent. As discussed in section two, these dynamics are inherently political, with the typology making visible how they play out across different operational approaches.

A foundational contribution to conceptualising nexus implementation is Howe's (2019) ‘bundles and arrays’ framework. In this model, bundles are defined as a ‘set of actions that deliberately target a group of people in order to have a greater impact on improving conditions for them’ across two or more nexus domains (Howe, 2019, p. 5). These bundles take several forms: sequential (such as emergency relief followed by recovery or livelihood support); simultaneous (such as school meals and civic education happening in the same place); repeated (such as predictable seasonal cash transfers); or integrated, seeking to support two or more nexus outcomes (such as cash‐for‐training initiatives that address humanitarian needs, support development, and foster peacebuilding). Arrays, in turn, refer to the broader strategic grouping of such bundles across a given setting or country programme (Howe, 2019).

While Howe's (2019) framework offers a valuable programme‐level lens for structuring and comparing interventions, the typology developed here extends the analysis to organisational strategies, institutional positioning, and the political and operational trade‐offs that shape implementation across contexts. It sheds light on why certain organisations lean towards integrated approaches, while others adopt more cautious, adaptive, or coordinated models. It also helps to explain why hybrid forms frequently emerge, particularly because the nexus is rarely implemented as an ideal arrangement, but rather as a negotiated, politically‐conditioned, and context‐bound practice.

**FIGURE 1:**
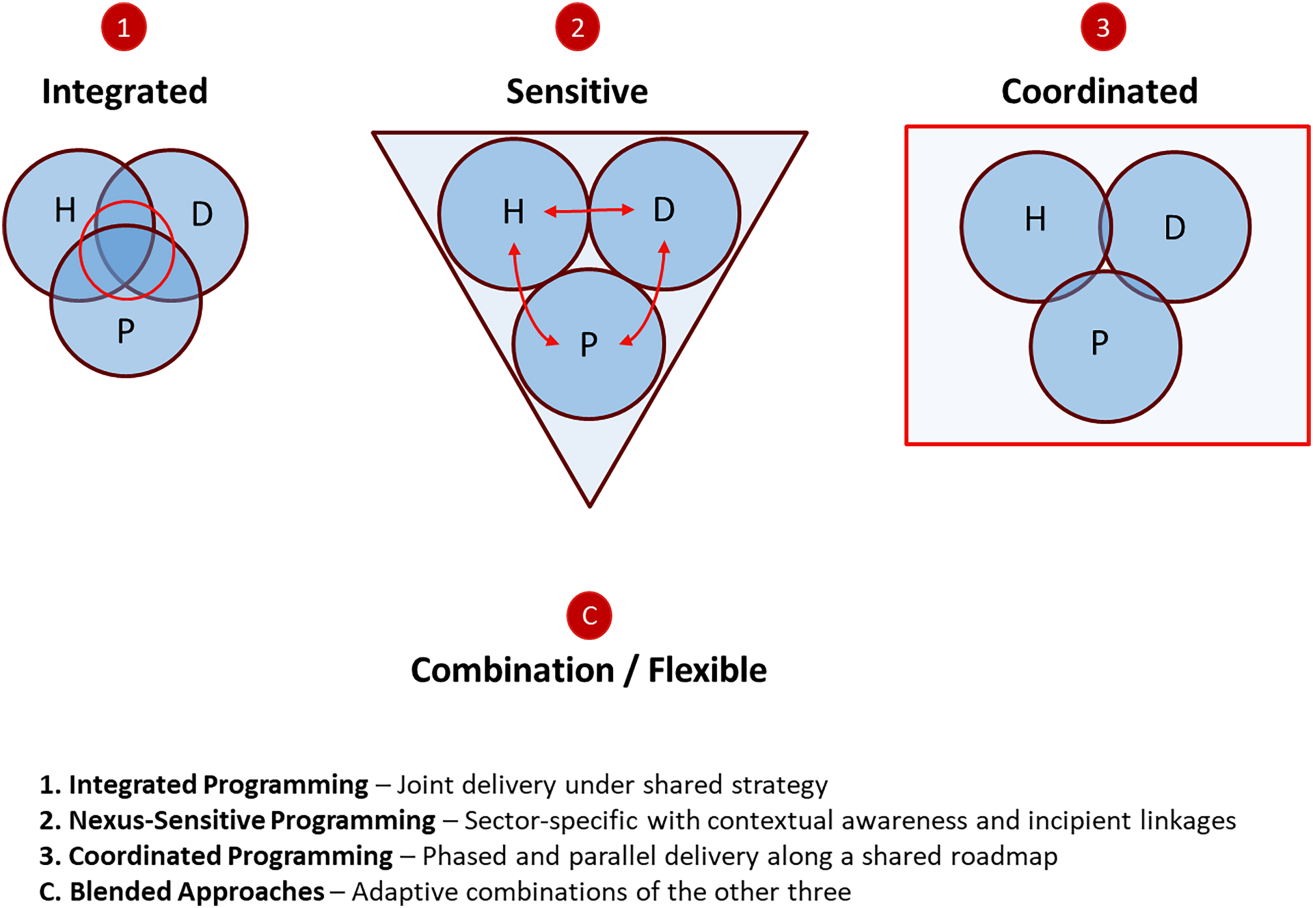
Typology of operational approaches to the triple nexus. **Source:** author.

Building on the methodology outlined above, this section introduces an operational typology of three dominant nexus modalities observed. These are not rigid categories or ideal types; rather, they represent recurring patterns in how organisations navigate humanitarian, development, and peace objectives under real‐world constraints. Figure 1 presents the typology visually, illustrating the three modalities and their areas of overlap. It highlights that nexus implementation is rarely linear or uniform. In practice, hybrid forms are common, as organisations combine elements of different approaches in response to shifting contexts, operational pressures, and political sensitivities.

Importantly, the typology does not simply distinguish between delivery modalities. Each approach reflects a particular negotiation between competing values and priorities and a different way of managing the tensions outlined in section two. As Südhoff (2025) and Wilson and Cochrane (2025) note, these lenses frequently collide during implementation, where ambitions for integration are constrained by fragmented institutional incentives, funding structures, time frames, accountability systems, and political realities.

Therefore, for example, integrated approaches may offer the greatest potential for coherence, but also present higher political and coordination risks. Nexus‐sensitive approaches provide flexibility and ethical safeguards, yet there is a risk of superficiality or fragmentation. Coordinated approaches strengthen alignment without forcing convergence, but they may reinforce sequential siloing or defer peace indefinitely. Viewed in this way, the typology responds to the ‘call for a more nuanced, overlapping mode’ of seeing the nexus (Plesner Volkdal, 2024, p. 147), while making visible the political, ethical, and structural tensions embedded in nexus practice. This creates an unavoidable tension: the typology introduces analytical categories, yet its purpose is not to standardise nexus practice, but to make visible the flexibility, overlap, and trade‐offs that shape it.

The following subsections introduce each of these modalities in turn. Section five expands on them and explores their challenges, opportunities, and operational dimensions.

### 4.1 Integrated programming: one plan, many lenses

Integrated programming represents the most ambitious form of HDP nexus operationalisation. It also reflects how the nexus is most commonly imagined: an approach that seeks to deliver jointly humanitarian, development, and peacebuilding activities under a single strategic umbrella (see, for example, CIC, 2019; OECD, 2019). This often entails shared assessments, joint planning processes, consolidated budgets, and unified monitoring and evaluation systems. Integrated models are typically pursued by multi‐mandate organisations or well‐aligned consortia capable of working across multiple sectors with a common analytical frame (Howe, 2019).

This integrated modality brings together what initially appeared to be distinct systems‐ and governance‐oriented approaches (respectively emphasising delivery architecture and institutional steering), illustrating how these logics can converge in practice within a single integrated nexus strategy.

The premise behind integrated programming is simple but powerful: crises are multidimensional, so responses must be too. The integrated model assumes that delivering relief, building resilience, and fostering peace are interdependent goals best achieved when pursued simultaneously and through collaborative delivery mechanisms.

For instance, the International Committee of the Red Cross (ICRC)'s intervention in the Donbas, Ukraine, during 2017–18 is a well‐cited early example found in the sources reviewed that can be classified as part of this approach. It combined technical efforts (water infrastructure protection), legal engagement (dialogue on international humanitarian law), and municipal partnerships to deliver public health gains while simultaneously reinforcing local governance and conflict mitigation (Schmitz Guinote, 2019). In this case, humanitarian, developmental, and peace objectives were not sequenced or siloed but pursued as co‐equal and mutually reinforcing.

The case further illustrates both the promise and difficulty of integration: coherence was achieved not simply by combining activities, but also through sustained legal, technical, and municipal engagement that required institutional alignment and careful political navigation.

The strength of integrated programming lies in its coherence. It enables actors to pool resources, avoid duplication, build systemic resilience rather than conduct isolated fixes, and adapt programming as needs change. One of the biggest challenges to this model is that integration requires high levels of institutional coordination, the alignment of diverse planning cycles, and the capacity to navigate potentially conflicting norms, especially around humanitarian neutrality. This makes integrated approaches both resource‐ and time‐intensive.

Depending on the type of peace programming, challenges in one component of an integrated nexus initiative can have ripple effects across the other two in this HDP type. This is particularly true for the peace dimension: some interviewees shared that externally‐imposed or donor‐driven agendas risk reinforcing existing power hierarchies and undermining local ownership and legitimacy. Moreover, integrated approaches tend to favour large, well‐resourced actors and may marginalise local or single‐mandate organisations that lack the structural bandwidth to participate fully in such models.

Yet, as Sommardahl (2025) notes in relation to Plan International's multi‐country children and armed conflict programme, maintaining such coherence necessitates constant conflict sensitivity, especially when navigating divisive or politically‐charged topics like child recruitment. At the same time, ‘frameworks for analysing conflict sensitivity of development, humanitarian or peace action can help provide a common language across the HDP nexus for talking about the negative and positive effects that programming has on conflict or conflict‐prone contexts’ (Sommardahl, 2025).

In practice, integrated programming is therefore most plausible where actors have compatible mandates, sufficient trust, and access to flexible funding; it becomes more difficult, however, where peace objectives are politically sensitive or where local actors are excluded from design.

### 4.2 Nexus‐sensitive programming: staying in lane, looking sideways

Where full integration is not feasible, owing to institutional or contextual constraints, organisations may adopt a nexus‐sensitive approach—in order, for instance, to have a humanitarian response that is ‘development‐sensitive’ (Howe, 2019, p. 7). This model recognises the interdependence of humanitarian, development, and peace dynamics but does not attempt to merge them into a single (organisational) delivery system. Instead, it encourages each actor or programme to remain ‘in its lane’ while being mindful of and responsive to the surrounding setting and to the work of other sectors, eventually developing incipient forms of coordination and support within them (Howe, 2019; Mena, 2025).

A case in point is Oxfam's work in South Sudan, which provided emergency assistance (such as cash transfers, borehole repairs, and livestock distribution) while also embedding governance dialogues and the promotion of gender equality (Fanning and Fullwood‐Thomas, 2019). These interventions were not integrated in a technical sense, but they were conceptually aligned with broader peace and development goals. This illustrates how nexus‐sensitive programming can create practical alignment without full institutional integration, relying instead on contextual analysis, informal coordination, and attention to potential spillovers across sectors.

Nexus‐sensitive programming entails a forward‐looking design that anticipates interactions and aims to maximise synergies while reducing harm (such as fuelling conflict, undermining neutrality, duplicating efforts, or creating dependencies) by avoiding negative spillovers from one domain into another. It does not necessitate joint funding or planning, but it does require awareness and some level of coordination. This approach is often suited to actors operating under strict mandates (such as humanitarian agencies concerned with neutrality) but which nonetheless recognise the importance of broader structural and political factors. This logic aligns with Wilson and Cochrane's (2025) insights from Ethiopia and South Sudan, where local actors navigate nexus tensions not through formal integration, but through informal, adaptive strategies attuned to political realities.

While it draws on similar analytical tools as conflict‐sensitive programming, nexus‐sensitive work is broader in scope: rather than focusing only on how interventions affect conflict dynamics, it considers potential interactions across humanitarian, development, and peace domains, including funding, mandates, institutional incentives, and long‐term trajectories (Sommardahl, 2025).

The strength of the nexus‐sensitive approach lies in its flexibility and contextual responsiveness. It can be adopted by actors with narrow mandates and still foster coherence. However, its limitations are equally significant. Without formal structures or shared metrics, the risk of superficiality is high. Sensitivity may become a discursive gesture rather than a genuine planning principle. Moreover, in the absence of ad hoc accountability mechanisms, sensitive programmes may fail to generate the sustained collaboration or systemic change to which the nexus aspires. Yet, as Kemmerling, Yıldırım‐Schlüsing, and Haidara (2025) show, even flexible and context‐sensitive approaches risk reproducing exclusion if they fail to engage critically with intersectional power dynamics, such as gendered cultural assumptions about who is considered a legitimate or neutral actor. These blind spots can ultimately reinforce inequality and undermine the inclusive objectives of nexus programming.

In essence, the sensitive approach is often the most realistic entry point for nexus work, but also the most fragile in practice. Reviewed cases indicate that many organisations adopt this model after failing to implement, or recognising the complexity of, integrated programming, or on being unable to fund all three components. For reasons of confidentiality and sensitivity, these cases are not detailed here, but a typical example would be a large humanitarian NGO shifting towards lighter coordination with development and peace entities after encountering operational barriers to full integration. This pattern also reflects how many actors initially pursue an integrated approach with limited awareness of alternative nexus models, with some informants suggesting that earlier awareness might have led to more feasible choices for their context or organisation.

In practice, this modality is useful where full integration would be politically or operationally unrealistic, but where actors still need to anticipate how their interventions affect, and are affected by, adjacent humanitarian, development, and peace dynamics.

### 4.3 Coordinated programming: multiple tracks, shared roadmap

The coordinated approach reflects a pragmatic response to the operational and political complexities of nexus implementation. Rather than aiming for simultaneous delivery or full integration, this model aligns humanitarian, development, and peacebuilding actions across time and space. It relies on strategic frameworks, such as theories of change, roadmaps, or collective outcomes, that guide different actors along a shared trajectory, even if their actions remain distinct (OECD, 2019; National HDP Nexus Task Force, Cameroon, 2023).

Document reviews and interviews revealed that coordinated programming is often the modality of choice in fragile or conflict‐affected settings where security conditions, government capacity, or funding structures challenge full convergence. It can accommodate different speeds of delivery, allowing humanitarian action to address urgent needs while laying the groundwork for more durable development or peace investments.

Cameroon's National HDP Nexus Task Force, established in May 2019, serves as a strong illustration. It convened humanitarian, development, and peace entities to develop a collective outcomes framework. While individual actors continued to deliver sector‐specific activities, their efforts were aligned via joint planning tools, monitoring systems, and periodic reviews (National HDP Nexus Task Force, Cameroon, 2022, 2023). This model preserved the autonomy of each component while fostering convergence in relation to common goals centred on longer‐term development and peace objectives. The case, as such, shows that a coordinated approach can create practical convergence without the merging of mandates, but it depends heavily on sustained convening power, shared monitoring, and continued political commitment.

The strength of coordinated programming lies in its realism. It allows for phased and context‐sensitive engagement and respects the distinct comparative advantages of different actors. Yet, the model is vulnerable to fragmentation if coordination is weak or if political will falters. The coordinated label itself can become problematic if peace or development goals are indefinitely postponed, creating an illusion of coherence when in practice there is drift or delay. It is also problematic in protracted conflict settings where two groups living near each other may have emergency relief and development needs, respectively.

Moreover, this approach often depends on strong convening power, meaning the ability to bring diverse actors together and coordinate their efforts, usually exercised by donors or multilateral agencies, which may privilege certain voices and marginalise others. As such, even when well‐designed, coordinated models must guard against reinforcing the very silos that the HDP nexus seeks to overcome and the power imbalances ever‐present in crisis contexts.

In practice, coordinated programming tends to be most useful where actors cannot or should not merge activities, but can still align planning, sequencing, and monitoring around shared outcomes.

### 4.4 Blending approaches: a typology for reflexive practice

This three‐fold typology—integrated, nexus‐sensitive, and coordinated—should not be viewed as fixed, but rather as heuristic lenses for reflection and guidance. In many responses, elements of all three may coexist. In the Donbas, for instance, the aforementioned ICRC programme combined integrated delivery with a sensitive posture towards shifting conflict lines. In Cameroon, coordinated planning is layered with locally‐integrated efforts where security permits. Few if any real‐world interventions reviewed in this research fit neatly within a single model, as illustrated in previous examples. Instead, and importantly, nexus strategies often evolve through the utilisation of a combination of approaches, shaped by context, capacity, and constraints. Integrated programmes may rely on sensitive components; coordinated frameworks may contain tightly sequential ‘islands’; and sensitive efforts may mature into more ambitious coalitions over time.

To clarify the trade‐offs involved, Table 1 summarises the conceptual and structural dimensions of each nexus modality. These include mandate scope, principles, degree of integration, or funding configuration, such as pooled funding (which refers to funding arrangements that combine resources across donors, sectors, or programme streams to support shared objectives, while multi‐year funding allows actors to plan beyond short project cycles).

**TABLE 1:** Summary of nexus approaches: distinguishing dimensions and trade‐offs.

Dimension	Approach		
	*Integrated*	*Nexus‐sensitive*	*Coordinated*
**Level of integration**	High (joint delivery)	Medium (context‐aware alignment)	Low to medium
**Organisational complexity**	High	Medium	Variable
**Risk to humanitarian principles**	High (depending on the peace programming)	Low	Low
**Funding model**	Blended/pooled	Single stream for each component	Multi stream
**Monitoring approach**	Shared metrics/outcomes	Impact‐aware	Agree shared roadmap across the nexus
**Main strength**	Holistic and a focus on synergies	Flexibility and autonomy	Clarity and coordination
**Main vulnerability**	High coordination cost/politicisation risk	Tokenism/fragmentation/lack of adaptability	Loss of momentum/over‐sequencing/reinforcing silos/lack of adaptability across sectors

**Source:** author.

Table 2 (introduced in section five) builds on these foundations to analyse how each modality navigates operational challenges, including funding rigidity, measurement, power dynamics, localisation, and peace sensitivities. Importantly, these dimensions should be understood as indicative, navigational tools rather than as definitive classifications, as there are examples that deviate from them.

**TABLE 2:** Summary of operational dimensions by challenge area.

Challenge area	Approach		
	*Integrated*	*Nexus‐sensitive*	*Coordinated*
**Conceptual clarity of peace**	High ambition, risk of politicisation especially if focused on peacemaking or peacekeeping	Moderately defined, limited scope	Often deferred or undefined
**Neutrality and principles**	Potentially compromised	Generally preserved	Strongly preserved, but limits synergy
**Timeline compatibility**	Requires coordination across cycles	Coexists with existing cycles	Chronologically staggered, but may not adequately address the needs of different communities
**Funding alignment**	Needs pooled/multi‐year funds	Adaptable to current systems	Often fits with existing donor logic; however, prioritisation accorded to emergency relief over development and peace
**Monitoring and measurement**	Shared metrics and overarching goal, difficult to manage	Limited metrics, qualitative proxies	Pillar‐specific metrics, with variable coherence

**Source:** author.

Table 1 further reinforces that each approach reflects distinct assumptions about risk, time, and power. Integrated programmes imagine cohesion and long‐term transformation, but demand high levels of coordination, shared trust, and institutional flexibility. Nexus‐sensitive work emphasises autonomy and context awareness, but it can risk superficial engagement with structural issues if not adequately resourced or supported. Coordinated models offer clarity and manageable coordination, but they can fall into inertia if peace is indefinitely deferred or development gains are repeatedly disrupted.

Importantly, this typology is also a tool for operationalising other agendas such as disaster risk reduction, localisation and Grand Bargain, or gender‐responsive programming (Barakat and Milton, 2020; Mena et al., 2022; Ansell, 2025; Kemmerling, Yıldırım‐Schlüsing, and Haidara, 2025; Wilson and Cochrane, 2025). Each mode creates different entry points: integrated programming embeds risk and resilience in its very design; nexus‐sensitive models use them as ethical and analytical lenses; and sequential coordination treats them as through‐lines for phased investment and shared accountability.

While reflecting current practice, the typology also invites critique. Nexus modalities are shaped by institutional politics, donor agendas, and geopolitics that set priorities, define ‘peace’, and value certain knowledge. Power dynamics and factors such as gender, race, class, and locality further influence how the triple nexus is experienced. These broader dimensions, although not the main focus here, will be addressed later and remain central to current debates.

As argued above, the HDP nexus should be understood as a politically‐negotiated space rather than as a neutral coordination model, creating an arena of practices (Hilhorst and Jansen, 2010; Hilhorst, 2018). Integrated models tend to concentrate power among large multilateral actors because they require centralised coordination and pooled funding, which can sideline local organisations. Nexus‐sensitive approaches create more conceptual and operational space for locally‐led strategies, but without structural mechanisms, they rarely shift decision‐making authority. Coordinated frameworks can offer openings for decentralised planning, yet unless convening power is genuinely shared, they may replicate donor‐led agendas and reinforce existing hierarchies.

As seen, each approach implies different assumptions, trade‐offs, and constraints. Recognising these ‘many shades’ not only helps practitioners to make informed decisions, but also surfaces the political, ethical, and structural tensions embedded in nexus work itself. This typology, then, is not merely descriptive; it also serves as a diagnostic tool to anticipate where each model is likely to thrive or encounter breakdowns, depending on political, institutional, and contextual conditions.

## 5 BETWEEN PROMISE AND PRACTICE: CHALLENGES AND OPPORTUNITIES ACROSS NEXUS APPROACHES

### 5.1 Conceptual and operational tensions

Ambiguity regarding peace, discussed above, affects each nexus modality differently. The various meanings are not merely semantic; they can shape operational choices and influence which nexus modality is seen as viable.

Integrated programming tends to foreground peace explicitly, which can draw actors into politicised framings, especially where peace is defined in state‐centric or securitised terms (Pedersen, 2016; Redvers and Parker, 2020). A common reference point is the ICRC's Donbas intervention, where infrastructure protection and legal dialogue were used to pursue public health gains while reinforcing governance and mitigating conflict dynamics through dialogue (Schmitz Guinote, 2019). Such integration can enhance coherence but may also raise neutrality concerns.

By contrast, nexus‐sensitive modality typically engages peace implicitly, emphasising do no harm and conflict sensitivity to avoid negative spillovers without claiming political influence. This preserves principled distance yet can slip into shallow engagement if not adequately resourced or linked to complementary efforts. Some scholars argue that community‐level peacebuilding, including social cohesion initiatives, can be essential for achieving humanitarian and development outcomes, cautioning against equating prudence with disengagement.

Coordinated approaches, which often unfold sequentially across time and space, frequently defer peace to later stages. This pattern has been criticised when exploring an ‘inverse nexus’, where peace is addressed alongside humanitarian or development programming, rather than only after the latter two components have stabilised the terrain (Mena and Hilhorst, 2022). While role delineation can reduce immediate tensions, over‐deferral risks rhetorical commitments that fail to materialise in concrete programming (Dalrymple and Swithern, 2019).

This ambiguity bleeds into operational choices, where peace is not only hard to define, but is also politically contentious and challenges its ‘boundaries with security and stabilisation policies’ (Südhoff, 2025). In short, ambiguity concerning what is ‘peace’, and whose definition prevails, cascades into modality selection, partnership configuration, and risk posture. It also explains why the same label (‘triple nexus’ or ‘HDP nexus’) masks divergent operational logics and political trade‐offs across settings.

These differences shape how actors position themselves in relation to peace: whether to engage explicitly with it despite political risks, to address it indirectly through conflict sensitivity, or to defer it within sequenced approaches.

### 5.2 Divergence of mandates, timelines, and planning cultures

As noted earlier, differences in sectoral mandates, planning cycles, and funding structures create systemic barriers to nexus implementation. For instance, humanitarian action typically operates according to short‐term logics, often measured in weeks or months, whereas development and peacebuilding have longer horizons (Poole and Culbert, 2019; Südhoff, Hövelmann, and Steinke, 2020). In practice, these divergences manifest differently across the three approaches under review:

- Integrated programming, while aspirationally coherent, faces practical constraints when funding cycles, organisational mandates, or log frames clash. Agencies may agree on collective outcomes but remain locked into divergent accountability systems.
- Nexus‐sensitive frameworks offer more temporal flexibility, yet they often lack the structural capacity to link short‐term relief to long‐term change.
- Coordinated approaches provide conceptual clarity through phase‐based planning, but they risk inertia when development or peace is repeatedly postponed due to prolonged crisis. This challenge is especially acute in protracted conflicts like those in Syria or Yemen, where the absence of peace frameworks has stalled any meaningful progression from relief to recovery (Mena and Hilhorst, 2022; Rey, Abellán, and Gómez, 2022).

This issue is even more complicated in contexts like South Sudan. Brown (2025) notes that national NGOs often shift mandates opportunistically, not as a strategic nexus alignment, but as a survival mechanism in a precarious funding ecosystem—similar dynamics have also been observed in the case of Yemen (see Mena and Hilhorst, 2022).

These misalignments can influence modality selection. Integrated approaches struggle most with the synchronising of planning cycles and mandates, which requires high institutional flexibility and funding continuity. Nexus‐sensitive frameworks adapt more easily to existing systems but may fail to build durable linkages. Coordinated models rely on sequencing to manage these differences, but risk reinforcing ‘handover thinking’, where development or peace is indefinitely delayed.

Financing further compounds these difficulties. Donors have increasingly signalled support for the nexus in rhetoric, but funding structures are still sectoral and inflexible (Dalrymple and Swithern, 2019; Rey, Abellán, and Gómez, 2022). This tension is reflected in the European Union's nexus approach, which, as Ansell (2025) points out, remains anchored in siloed planning despite rhetoric of integration under funding mechanism like the ‘Global Europe: Neighbourhood, Development and International Cooperation Instrument’. Peacebuilding (particularly in its community‐based or justice‐oriented forms) is rarely financed on the same scale or timeline as humanitarian or development work (Brown, Mena, and Brown, 2024; Sommardahl, 2025).

Integrated approaches typically require pooled or multi‐year funding, yet few donors are willing to invest in joint mechanisms that blur traditional categories. Nexus‐sensitive frameworks are somewhat more adaptable to existing funding streams but often struggle to mobilise sufficient resources for forward‐looking or preventive components. Coordinated models, while aligned with donor structures, risk reinforcing silos if funding is not synchronised across sectors or if handovers are under‐resourced.

### 5.3 Structural asymmetries and political stakes

Beyond the conceptual and structural challenges outlined, measurement and evaluation present another persistent tension. As Sommardahl (2025) shows, conflict sensitivity indicators (when embedded in humanitarian and development pillars) can help to tackle this issue, but they require rigorous adaptation and flexible MEL systems. Importantly, conflict sensitivity should arguably be part of peace programming as well. Labelling it as the ‘P’ in the triple nexus can be problematic, as conflict sensitivity does not necessarily lead to a more peaceful society; rather, it aims to make programming more effective and context‐appropriate. While development and humanitarian sectors have developed robust metrics for performance and impact, peace interventions present less standardised indicators. This can create difficulties not only for accountability but also for learning and adaptation (Howe, 2019; Barakat and Milton, 2020; Südhoff, 2025). To address these challenges, Plesner Volkdal (2024) proposes HDP evaluation and analytical frameworks that offer indicators across three dimensions: resilience; conflict reduction; and social protection. This approach seeks to enable a context‐sensitive yet comparable evaluation of HDP initiatives, linking theory to practice through measurable metrics and case‐based application.

Translating these measurement and evaluation challenges to the typology, integrated programmes, which rely on joint monitoring frameworks, face the difficulty of defining common indicators that capture humanitarian, development, and peace effects simultaneously—a demanding and sometimes politically‐sensitive task. Nexus‐sensitive approaches tend to use qualitative or proxy indicators rooted in contextual analysis, but these often fail to capture cross‐sector effects. Coordinated models usually retain sector‐specific MEL systems, which protect accountability but may miss cumulative or combined impact.

### 5.4 Navigating power and localisation across nexus modes

This subsection turns to how power and localisation play out across nexus modalities, building on the political dynamics discussed above. The HDP nexus, particularly in its integrated and policy‐driven forms, is often criticised as donor‐led, conceived in “Western’ capitals and multilateral boardrooms, and then retrofitted for local contexts (Brown, Mena, and Brown, 2024), raising concerns about the instrumentalisation of local actors, especially when definitions of peace align with geopolitical or security agendas (Nguya and Siddiqui, 2020; Steinke, 2021).

Humanitarian organisations warn that such alignment can compromise neutrality, particularly when nexus frameworks serve stabilisation or state‐building priorities. While nexus‐sensitive approaches may better respect local agency, without genuine inclusion they risk becoming performative localisation (Barakat and Milton, 2020), and coordinated strategies, although often claiming ‘country ownership’, are frequently driven more by donor timelines than participatory processes.

A particularly acute tension lies in the promise versus practice of localisation. While the triple nexus is often framed as enabling local ownership, in reality, its implementation is frequently shaped by external priorities, risk aversion, and hierarchical funding chains (Barakat and Milton, 2020; Torres and Dela Cruz, 2021; Khan, 2024). Meaningful localisation within nexus work requires more than participation, it demands shifts in decision‐making power, resourcing, and epistemic authority (Barakat and Milton, 2020). Furthermore, these dynamics are inseparable from the broader operational trade‐offs that different nexus approaches entail, which are explored in the next subsection.

### 5.5 Comparative operational dimensions and strategic implications

To illustrate how these differences play out operationally, Table 2 synthesises the key operational dimensions of the three nexus approaches. Building on Table 1's conceptual and structural distinctions, it shows how each modality navigates challenges such as neutrality risks, funding rigidity, localisation, power dynamics, and sequencing.

Rather than presenting fixed models, this comparison highlights the trade‐offs inherent in each form, revealing how issues such as mandate tensions and peace‐related sensitivities unfold differently depending on the chosen approach. The table should be read as indicative to support critical reflection, not a template for replication.

Despite these challenges, the HDP nexus offers important opportunities when treated not as a rigid model but as a framework for reflexive and adaptive practice. Integrated programmes, although demanding, can deliver holistic outcomes when grounded in context‐specific analysis and power‐sensitive engagement. Nexus‐sensitive programming allows actors to remain within their ethical mandates while contributing to shared goals, and coordinated programmes provide a pragmatic structure for multi‐actor collaboration where joint programming is not yet feasible.

Across all three approaches, the triple nexus enables risk‐informed planning, systems thinking, and stronger links between local and global knowledge. Yet, as Wilson and Cochrane (2025) caution (see also Cochrane and Wilson, 2023), without inclusive design such programming may provoke social tensions, as in cases where abrupt shifts from relief to development have triggered unrest. Realising these opportunities requires moving beyond technical fixes to acknowledge the inherently political nature of the HDP nexus, which reflects choices about what kinds of peace are possible, whose suffering is prioritised, and which futures are imagined. Recognising these tensions does not undermine the nexus but makes its application more honest and accountable.

These deeper stakes are not only about system efficiency, but also concern who gets to shape aid priorities, whose voices define peace, and how the triple nexus interacts with power asymmetries at the global, national, and local levels. The article turns to these structural and intersectional debates next, surfacing tensions around the expansion of the nexus, epistemic inequality, and injustices, as well as the nexus' blind spots with respect to gender, climate, and civic space.

## 6 LOCATING THE HDP TYPOLOGY WITHIN CURRENT DEBATES: POWER, INTERSECTIONALITY, AND POLITICS

This section, rather than expanding the HDP typology, situates it within contemporary debates that influence how nexus work is understood and experienced in practice. Instead of treating issues such as climate change, migration, or localisation as additional ‘pillars’, it shows how these concerns are embedded within the nexus and are experienced differently depending on whether integrated, nexus‐sensitive, or coordinated approaches are adopted. It therefore shifts attention from operational alignment alone to broader questions about what the nexus includes, who drives its implementation, and how it interacts with agendas such as localisation, decolonisation, climate action, and displacement.

In this context, intersectionality refers to how overlapping axes of power, including gender, race, class, and locality, shape both who benefits from nexus programming and who bears its risks, as well as the normative assumptions underpinning which forms of peace and development are prioritised.

### 6.1 Expanding or integrating the nexus?

A growing strand of literature has raised questions about the potential extension of the HDP nexus beyond its tripartite configuration. Two key proposals have emerged in this regard: the incorporation of anthropogenic climate change and the integration of human mobility and displacement as additional components of the nexus.

The case for including climate change stems from its perceived role in exacerbating both humanitarian emergencies and longer‐term development needs. Proponents argue that climate‐related hazards intensify vulnerabilities, displace populations, and can even amplify drivers of violent conflict (Abitbol and McCandless, 2022; IPCC, 2022; Mena et al., 2022; See, Opdyke, and Banki, 2026). While this is valid, critical reviews caution against isolating climate change as a standalone ‘fourth pillar’. Climate change, while undoubtedly consequential, does not independently cause disasters or conflict; instead, it acts as a multiplier within systems already characterised by vulnerability, inequality, and poor governance (Wisner et al., 2004; Kelman, 2020). This reinforces the argument for integrating climate change adaptation and disaster risk reduction into the existing HDP nexus rather than creating parallel, siloed streams (Mena et al., 2022). Such integration would make climate considerations more explicit across nexus responses while aligning them with existing international frameworks such as the Sendai Framework for Disaster Risk Reduction 2015–2030, the Paris Agreement, and the SDGs.^1^

Similarly, some actors propose adding migration and displacement as a fourth component of the nexus. This suggestion arises from recognition that forced migration, whether linked to conflict, disasters, or socio‐political instability, cuts across humanitarian, development, and peace domains (Nguya and Siddiqui, 2020; See, Opdyke, and Banki, 2026). Yet, much like the case for climate change, evidence and operational logic indicate that migration‐related concerns are already embedded within the triple nexus in practice. Rather than expanding the nexus, it may be more effective to clarify and enhance how migration is addressed within its existing streams (Burrows and Kinney, 2016; Rey, Abellán, and Gómez, 2022). This integrated approach would support durable solutions by linking immediate protection with long‐term development strategies for displaced populations and host communities alike (Macrae et al., 1997; Steinke, 2021).

For the typology, these debates matter because thematic expansion interacts differently across models. Integrated approaches offer the greatest potential to embed climate resilience or migration dynamics systemically, but they also risk overload and agenda inflation. Nexus‐sensitive approaches can integrate climate and displacement selectively through context analysis, but they often lack the structural influence to shift wider planning systems. Coordinated approaches can phase climate and migration issues over time, yet they may reinforce sequencing that postpones preventive or long‐term adaptation.

Moreover, these debates point less to the need for a new structural model and more to how existing types—integrated, nexus‐sensitive, and coordinated—incorporate cross‐cutting themes. Climate and displacement considerations may be embedded differently across the three approaches, with integrated programmes offering the most potential for systemic inclusion, nexus‐sensitive frameworks enabling targeted adjustments, and coordinated models addressing climate and displacement considerations through phased interventions. In this sense, the typology provides a framework to assess whether such integration strengthens coherence or risks creating new silos.

### 6.2 Intersectional complexities and internal organisational barriers

As discussed in subsection 4.4, localisation debates already highlight the concentration of decision‐making power in donor capitals and multilateral institutions. This subsection turns inward, focusing on how intersectional inequities, institutional cultures, and organisational structures shape the HDP nexus from within, and how these internal dynamics intersect with broader structural imbalances.

A persistent critique is that despite its collaborative rhetoric, the triple nexus remains a donor‐driven project shaped by the priorities of the so‐called Global North (Steinke, 2025). For actors working in fragile or conflict‐affected contexts, the neat categories of humanitarian, development, and peace work often fail to align with operational realities. Organisational silos, rigid hierarchies, and limited cross‐sector planning capacity further undermine coherence, while outdated structures, high staff turnover, and weak knowledge management systems prioritise donor compliance over collective outcomes (Steinke, 2021; Khan, 2024; Plesner Volkdal, 2025). These constraints may make integrated models the hardest to sustain, push many actors towards nexus‐sensitive arrangements as a compromise, and leave coordinated strategies as the most feasible option for siloed organisations, albeit at the cost of synergy.

Weak institutional learning compounds these barriers. Despite numerous pilots and policy commitments, few agencies have mechanisms to transfer lessons across sectors or settings. Short funding cycles and staff churn erode institutional memory, while shrinking civic space and authoritarian backlash may reduce opportunities for civil society engagement and critical feedback. Across the typology, these deficits limit adaptation: integrated programmes miss opportunities to refine joint delivery, nexus‐sensitive frameworks struggle to deepen synergies, and coordinated models fail to adjust sequencing based on evidence.

Poorly‐managed transitions between humanitarian relief and development or peace programming can cause real harm. Wilson and Cochrane (2025) document how in Ethiopia, abrupt operational shifts and uneven resource distribution between groups have fuelled tensions and, in some cases, conflict. In South Sudan, donor mistrust of state institutions and fears of corruption have resulted in centralised funding channels dominated by international actors, relegating national NGOs to subcontracting roles. As Kemmerling, Yıldırım‐Schlüsing, and Haidara (2025) note, such arrangements marginalise local gendered knowledge and reinforce top‐down decision making.

The same logics that concentrate power globally are often reproduced internally and locally, sidelining those with the contextual expertise and relationships needed for effective programming. In Ethiopia and South Sudan, these patterns have fuelled tensions, marginalised local expertise, and reinforced centralised control. More broadly, nexus approaches that overlook local dynamics, ignore political sensitivities, or bypass inclusive consultation risk exacerbating inequalities and undermining trust among affected populations (Cochrane and Wilson, 2023; Plesner Volkdal, 2024). What begins as an attempt at synergy may, if poorly managed, devolve into fragmentation or exclusion if care is not taken.

These are not isolated implementation weaknesses but symptoms of a deeper tension between the nexus' aspiration for integration and its embeddedness in systems still governed by competition, control, and unequal power. Addressing them demands more than technical fixes: it requires structural reform, organisational introspection, and a redistribution of decision‐making authority and resources to local actors and affected populations.

These observations reinforce a central argument of the article: the HDP nexus is not just a matter of coordination, but also of power, politics, and institutional positioning. Rather than expanding it to include ever‐more themes, the typology helps practitioners and researchers to assess how cross‐cutting agendas (such as climate change, equity, and localisation) are taken up, diluted, or politicised under different nexus modalities. In this way, rather than widening the nexus concept, these reflections deepen understanding of it.

## 7 CONCLUSION: A FLEXIBLE FRAMEWORK FOR COMPLEX REALITIES

This article has argued that the HDP nexus should not be understood as a singular or standardised model, but rather as a field of practice shaped by divergent mandates, political contexts, institutional logics, and embedded power relations. Through the proposed typology of integrated, nexus‐sensitive, and coordinated approaches, the analysis has shown how the triple nexus can be operationalised in practice through multiple, often hybrid, configurations. These are not fixed templates, but context‐specific responses to trade‐offs between ambition and risk, ethics and pragmatism, and autonomy and alignment.

This article's core contribution, therefore, is to shift nexus analysis from a primarily technical question of programme design towards an institutional and political question of how different actors interpret, negotiate, and operationalise nexus ambitions under real constraints. It takes the debate beyond ‘what does the nexus look like?’ to ‘what kind of nexus is possible, for whom, and under which conditions?’

By amending the analytical focus, the typology builds on and extends Howe's (2019) ‘bundles and arrays’ framework, moving from project‐level configurations to the systemic and political conditions that shape nexus practice.

A central conclusion is that nexus implementation cannot be reduced to a single model, and that any attempt to typologise it must remain attentive to the tension between analytical categorisation and the need to resist standardisation. Implementation varies by context, actor, mandate, and moment, as well as by the intensity and phase of conflict. As Howe (2019) suggests, in active conflict settings, actors may gravitate towards nexus‐sensitive approaches that preserve principled distinctions and manage political risk, while more stabilised or transitional settings may afford greater scope for integrated or coordinated models. In practice, humanitarian, development, and peace dynamics rarely unfold sequentially or predictably; instead, they overlap and interact in ways that can complicate planning and coordination.

This diversity has important implications for policy and practice. Efforts to standardise nexus programming through guidance or funding instruments risk constraining the very flexibility that makes nexus work viable on the ground. In this sense, it is more accurate to speak of *HDP nexuses*, in the plural. This insight is especially critical in a context of shrinking aid budgets and rising expectations that the nexus will deliver efficiency gains and ‘do more with less’. As the analysis reveals, the gap between conceptual enthusiasm and operational reality remains significant, forged by institutional incentives, power asymmetries, and political constraints.

Rather than promoting a preferred model, the typology offers a diagnostic tool to help actors assess when integrated, nexus‐sensitive, or coordinated approaches are more feasible, legitimate, or politically acceptable. It also highlights the risks associated with each: integration models may maximise coherence but heighten political exposure; nexus‐sensitive models safeguard principles but risk superficiality; and coordinated models may provide pragmatism while reinforcing silos or deferring peace. Making these trade‐offs explicit is central to the typology's practical value.

Lastly, the analysis cautions against folding additional agendas such as climate change, displacement, gender equity, or localisation into the nexus without sufficient clarity or accountability. These issues are not external to the nexus; they are embedded within it and taken up differently across modalities. The typology helps to determine whether such integration deepens coherence or risks conceptual overload.

Ultimately, the future of nexus practice depends not on expanding its scope, but on deepening its implementation—*prioritising depth over breadth*. This requires adaptive leadership, institutional reflexivity, and a willingness to confront the political and ethical stakes embedded in coordination. Rather than asking how to ‘apply’ the HDP nexus universally, we must ask: what does coherence look like here, now, and for whom? Framed in this way, the typology supports not only better coordination, but also better decision making by foregrounding the trade‐offs, risks, and implications associated with different forms of nexus engagement.

## CONFLICT OF INTEREST STATEMENT

The author declares that there are no conflicts of interest related to the research, authorship, or publication of this article.

## FUNDING STATEMENT

This work was supported by the European Research Council under the Horizon 2020 research and innovation programme (grant agreement: 884139).

## Data Availability

The data that support the findings of this study are available on request from the corresponding author. The data are not publicly available due to privacy or ethical restrictions.

## Endnote

^1^
Howe (2019) provides a detailed analysis of how the HDP nexus supports the achievement of the SDGs.
